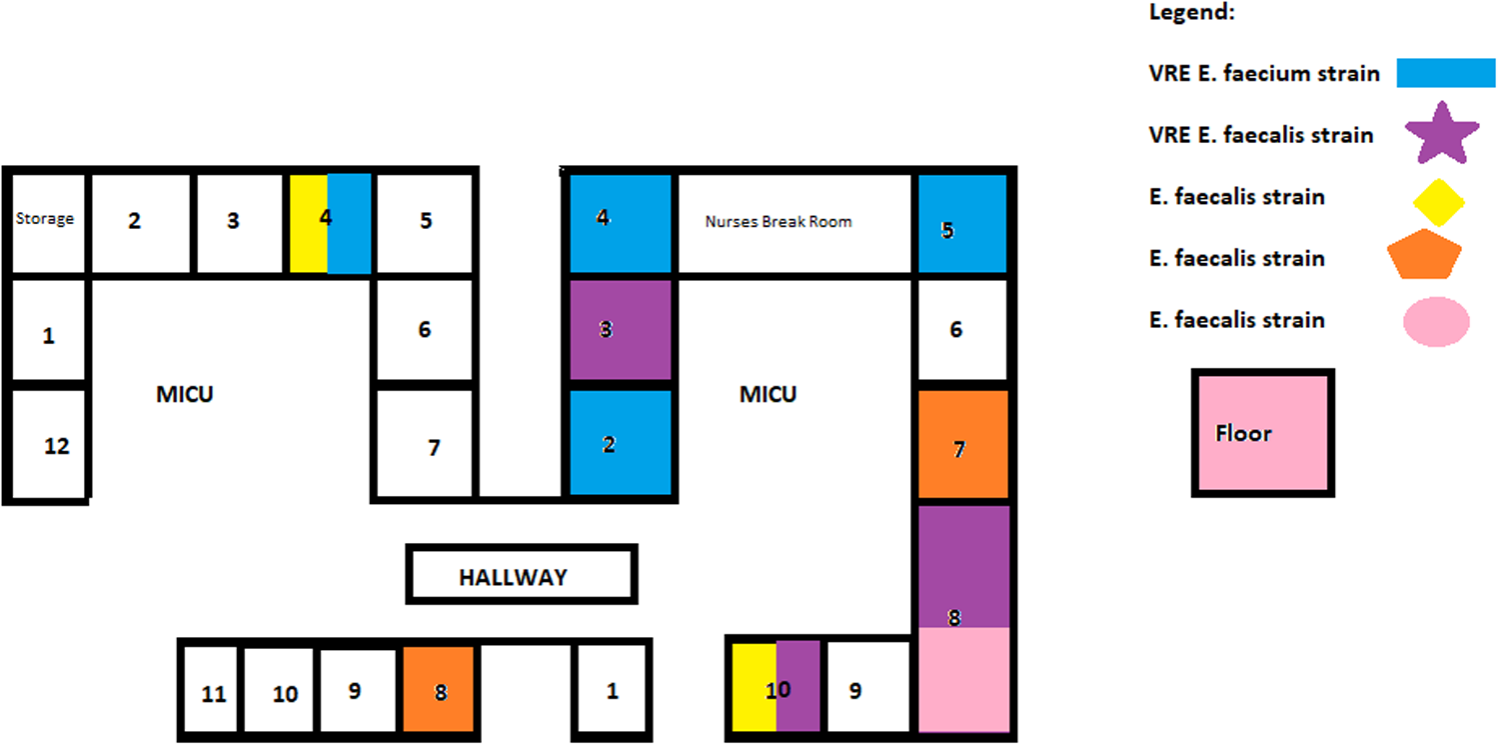# Enterococcal Bacteremia outbreaks during SARS-COV-2 in Intensive Care Unit (ICU): The role of Strain Identification

**DOI:** 10.1017/ash.2024.282

**Published:** 2024-09-16

**Authors:** Sharanjeet K. Thind, Ghias Sheikh, Syeda Sahra, Dena R. Shibib, Awais Bajwa, Houssein A. Youness, Christopher Gentry

**Affiliations:** Mercy Hospital, Oklahoma City; OUHSC; University of Oklahoma Health Sciences Center

## Abstract

**Background:** An unprecedented burden of morbidity and mortality has been reported in patients admitted to healthcare facilities with SARS-COV-2 infection globally since March 2020. A higher incidence of ICU-acquired bloodstream infection has been described with the cumulative risk increased with the length of ICU stay, use of steroids, anti-inflammatory agents, and indwelling catheters. Additionally, SARS-COV-2 infection may increase the risk of bacteremia from gastrointestinal flora such as Enterococcus species by disrupting the gastrointestinal barrier and microbiome. **Methods:** We aimed to investigate the outbreaks of Enterococcus bacteremia in patients with SARS-COV-2 infection and included patients aged>18 years admitted to the ICU at the Oklahoma City Veterans Affairs Medical Center who were infected with SARS-COV-2 and were identified as having positive blood cultures for Enterococcus faecalis, Enterococcus faecium including vancomycin-resistant species and other bacterial or candida species between March 1, 2020, to March 9, 2022. We collected the following data: duration of hospitalization including ICU stay, unit and room location during the hospital stay, blood culture collection date and results, duration, and site of central line placement, duration of ventilation, administration of antimicrobials and COVID-19 directed therapeutics using computerized patient record system (CPRS). We sent 10 E. faecalis and 12 E. Faecium isolates for genetic analysis. DNA was sequestered from each isolate and multi-locus sequence typing (MLST) was performed by amplifying 7 regions of the E. faecalis genome and 7 regions of the E. faecium genome by polymerase chain reaction (PCR). Resulting amplicons were sequenced and allele type for each gene region and overall sequence type was determined using MLST database (http://pubmlst.org). **Results:** There were 22 episodes of enterococcal bacteremia in a 3-month duration in the ICU in 20 patients of which 17 were associated with another episode with the same strain (Figure-1-2). Central line placement was noted in 18/22 episodes. Genetic analysis by multi-locus sequence typing of enterococcal bacterial strains was performed by the Public Health Reference Laboratory, Palo Alto. Similar strains were localized to patients in the same geographical region in the ICU (Figure 3). The isolates from 2 other patients who presented with the same strain of Enterococcus but were never hospitalized at the same time during the COVID-19 pandemic were localized to another hospital where both had received care at different times. **Conclusion:** Higher rates of enterococcal bacteremia were reported during the SARS-COV-2 pandemic. Geographical proximity with strained infection control measures accounted for ICU outbreaks seen in our tertiary care setup.

**Figure:**
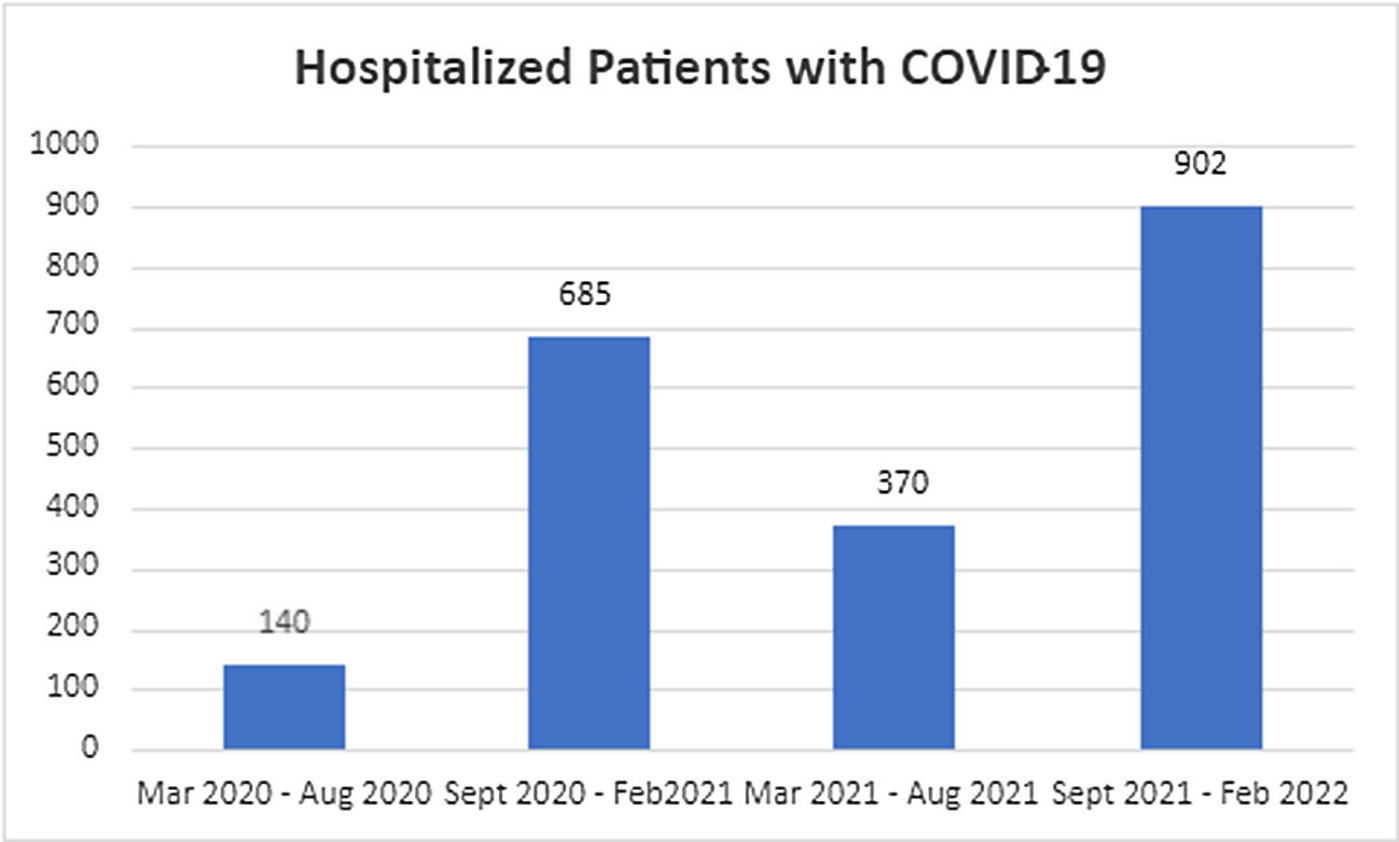

**Figure:**
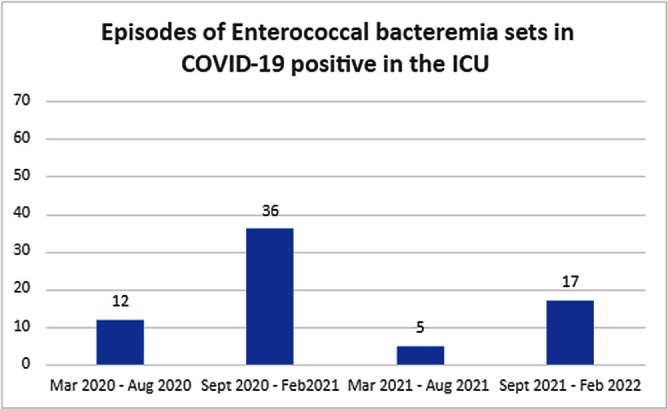

**Figure:**